# Supplementary material for: A substrate-driven allosteric switch that enhances PDI catalytic activity
Source: Nat Commun. 2016 Aug 30;7:12579. doi: 10.1038/ncomms12579 (PMC5013553; doi:10.1038/ncomms12579)
Supplement: Supplementary Information — Supplementary Figures 1-9, Supplementary Table 1 and Supplementary Methods. [file ncomms12579-s1.pdf]

Supplementary Figures

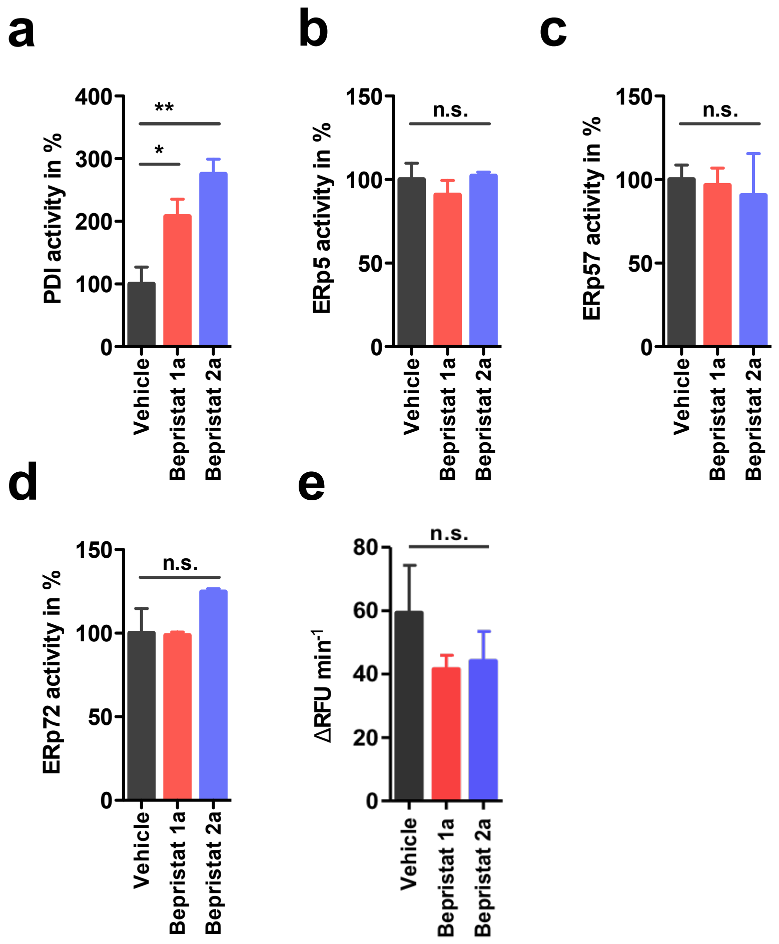

Supplementary Figure 1. **Selectivity of the bepristat-mediated augmentation of di-eosin-GSSG reductase activity.** (a) The effect of 30 μM bepristats on di-eosin-GSSG cleavage was evaluated for full-length PDI (n=3) and augmentation was quantified. In contrast, bepristats did not augment activity of (b) ERp5 (n=3), (c) ERp57 (n=3), (d) ERp72 (n=3) in the same assay. (e) When no enzyme was added, bepristats failed to augment the cleavage of di-eosin-GSSG by DTT (n=4). Lack of activity of the bepristats when used with other thiol isomerases demonstrates the selectively of their augmenting effect on PDI catalytic activity. Data are mean $\pm$ SEM from three independent experiments. One-way ANOVA with Dunnett's post test: *p < 0.05; **p < 0.01; n.s., non significant.

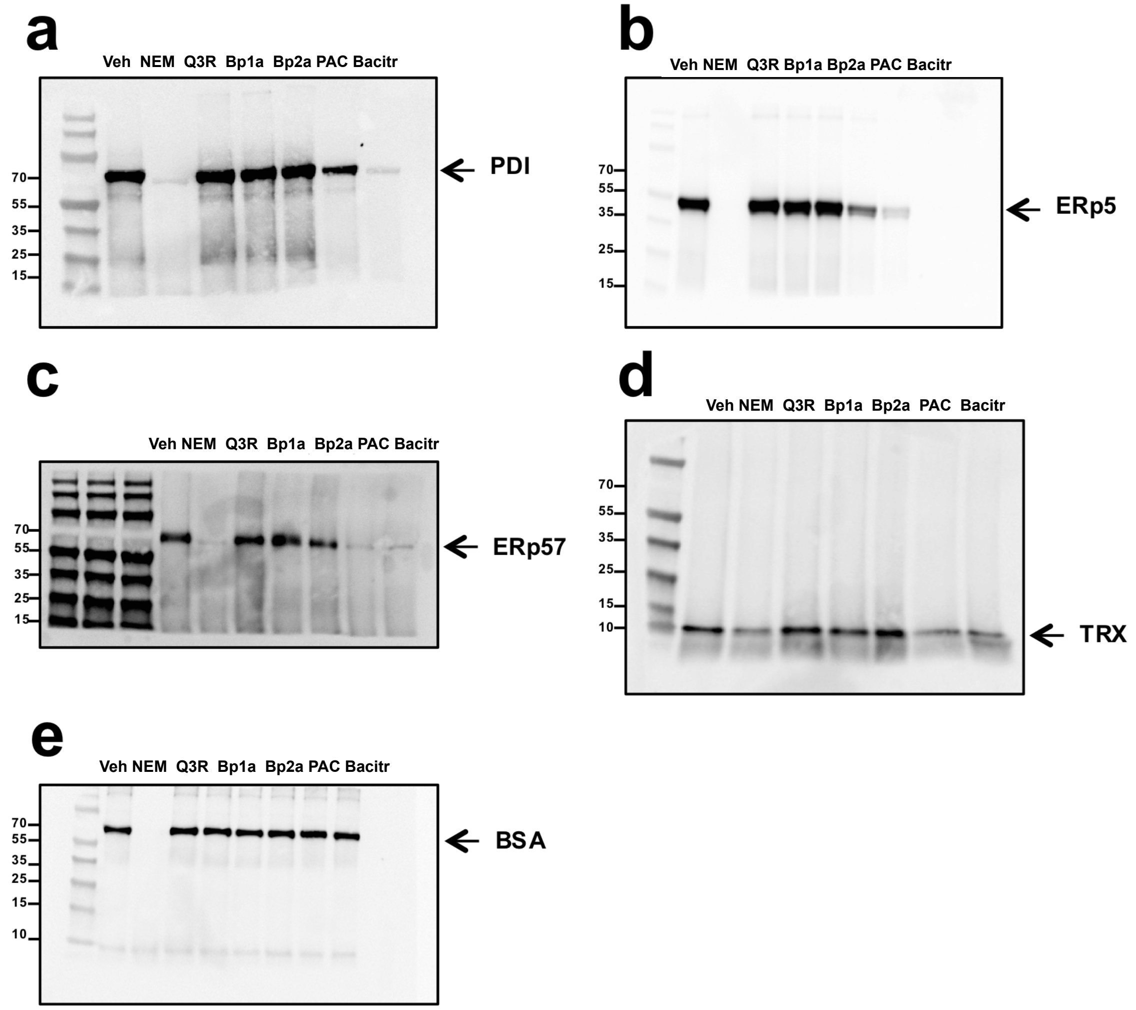

Supplementary Figure 2. Original images of representative immunoblot of MPB-binding (n=3-6) of PDI (a), ERp5 (b), ERp57 (c), TRX (d) and BSA (e) as shown in figure 2C. Results using vehicle (*Veh*), N-ethylamine (*NEM*), quercetin-3-rutinoside (*Q3R*), bepristat 1a (*Bp1a*), bepristat 2a (*Bp2a*), PACMA-31 (*PAC*), and bacitracin (*Bacitr*) are shown.

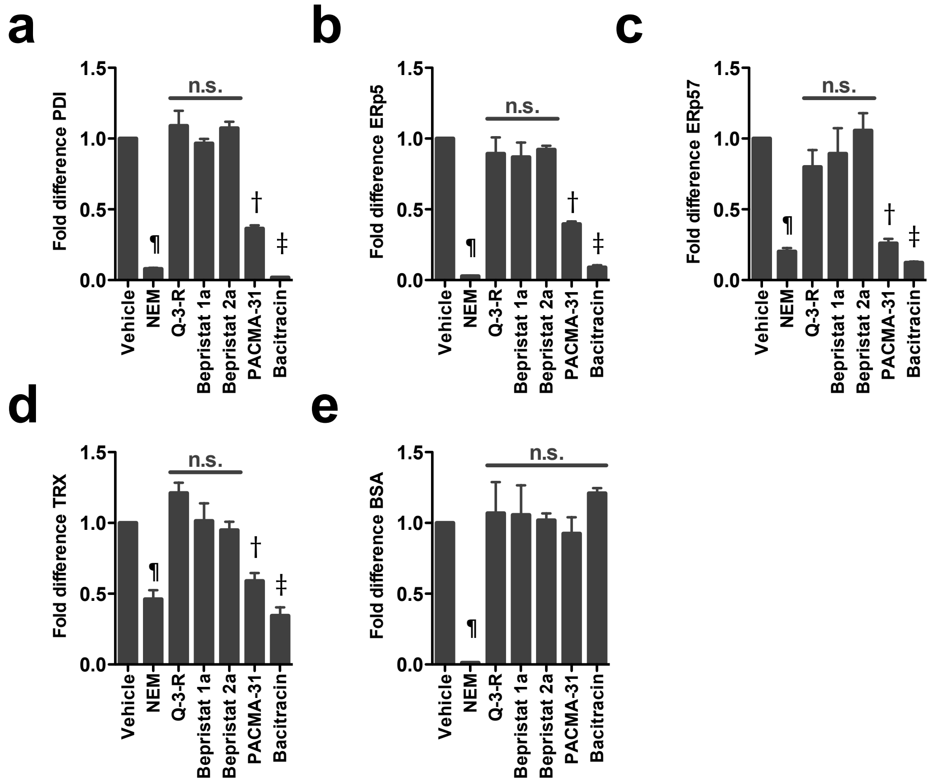

Supplementary Figure 3. Bacitracin and PACMA-31 non-selectively bind to free sulfhydryls on thiol isomerases. (a) PDI, (b) ERp5, (c) ERp57, (d) thioredoxin, or (e) BSA were incubated with vehicle, 300 µM NEM, 150 µM quercetin-3-rutinoside, 150 µM bepristat 1a, 150 µM bepristat 2a, 150 µM PACMA-31 or 150 µM bacitracin prior to exposure to MPB. Samples were then analyzed by SDS-PAGE and MPB was detected by Cy5-labeled streptavidin. Fluorescence corresponding to MPB binding was quantified using ImageQuant 400 analysis software. Values represent mean fluorescence ± SEM (n = 3-6). One-way ANOVA with Bonferroni's post test: ¶ $p < 0.001$, NEM vs bepristat 1a, NEM vs bepristat 2a; † $p < 0.05$, PACMA-31 vs bepristat 1a, PACMA-31 vs bepristat 2a; ‡ $p < 0.05$, bacitracin vs bepristat 1a, bacitracin vs bepristat 2a; n.s.,non significant.

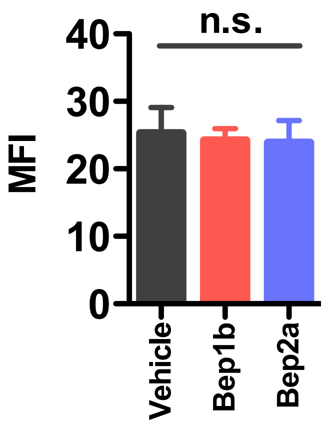

Supplementary Figure 4. **Bepristats do not affect P-selectin expression.** P-selectin expression in response to 2 μM of the PAR-1 activating peptide, SFLLRN, was monitored by flow cytometry following exposure of platelets to vehicle control (*black*) or 45 μM bepristat 1b (*red*) or bepristat 2a (*blue*). Values represent mean $\pm$ SEM from three independent experients. One-way ANOVA was performed, showing no significant difference between the three conditions ($p = 0.84$).

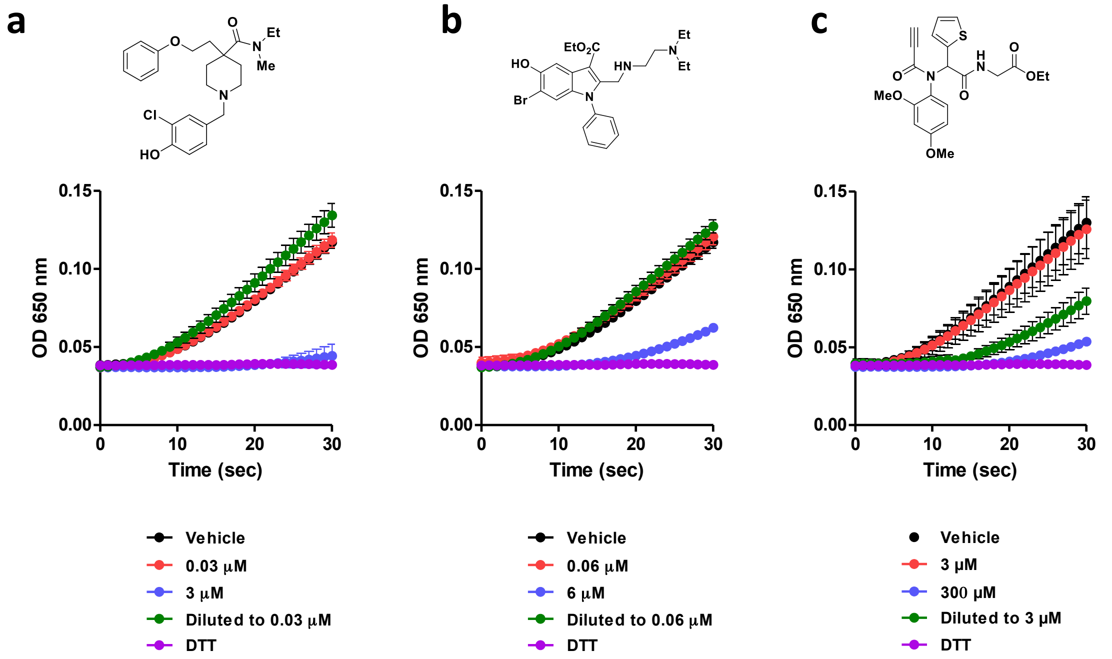

Supplementary Figure 5. Reversibility of bepristats. Reversibility of inhibition of reductase activity of recombinant PDI by either (a) bepristat 1a, (b) bepristat 2a, or (c) PACMA-31 was evaluated by 100-fold dilution of a mixture of 3 µM bepristat 1a, 6 µM bepristat 2a or 300 µM PACMA-31 and monitoring in the insulin turbidimetric assay. Values represent mean data + SEM.

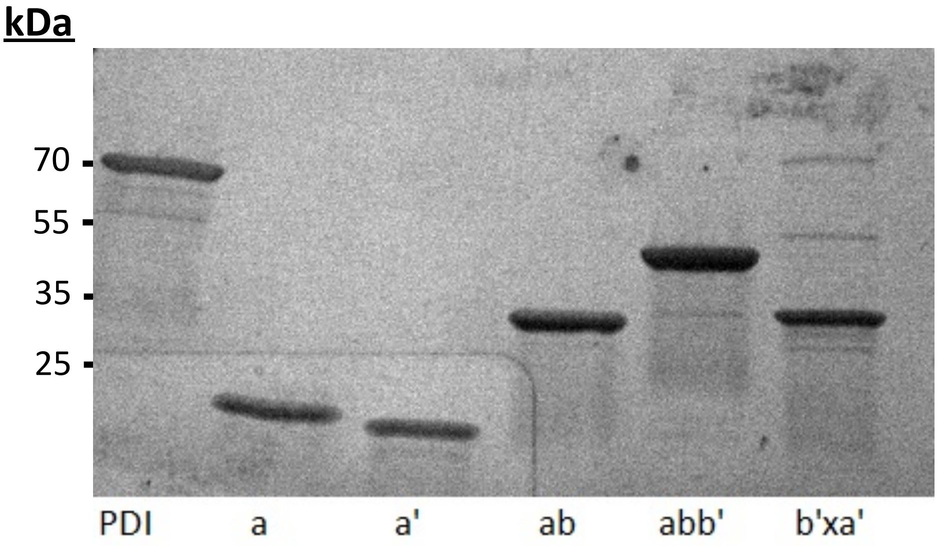

Supplementary Figure 6. **PDI fragments.** SDS-PAGE analysis of PDI and PDI fragments used in these studies.

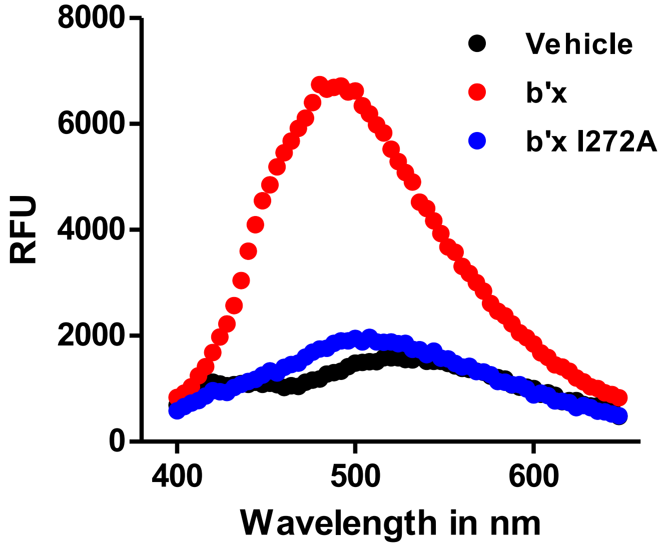

Supplementary Figure 7. **ANS fluorescence is blocked in the I272A mutant.** ANS was incubated with either vehicle alone, wild-type b'x domain, or a b'x domain mutant in which isoleucine 272 was mutated to alanine. The I272A mutation inhibited ANS fluorescence.

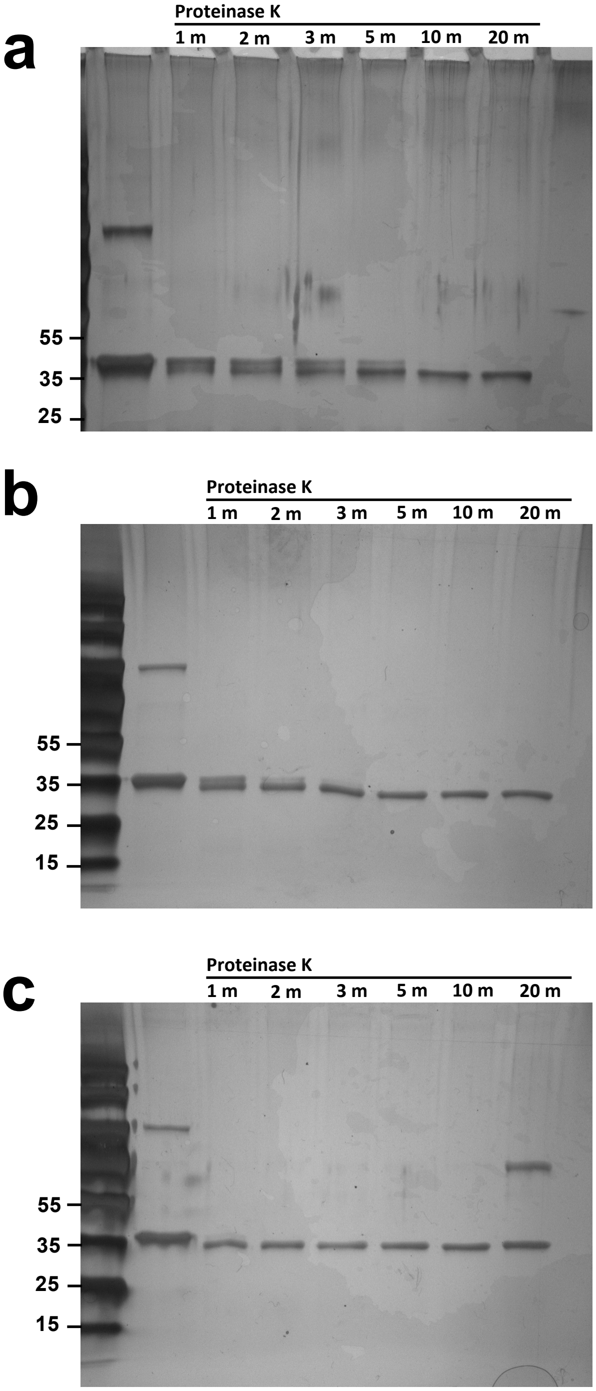

Supplementary Figure 8. Original images of representative silver stained gels of abb'x following proteinase K digestion (n=3 for all conditions) in the presence of vehicle (a), bepristat 1a (b) or bepristat 2a (c).

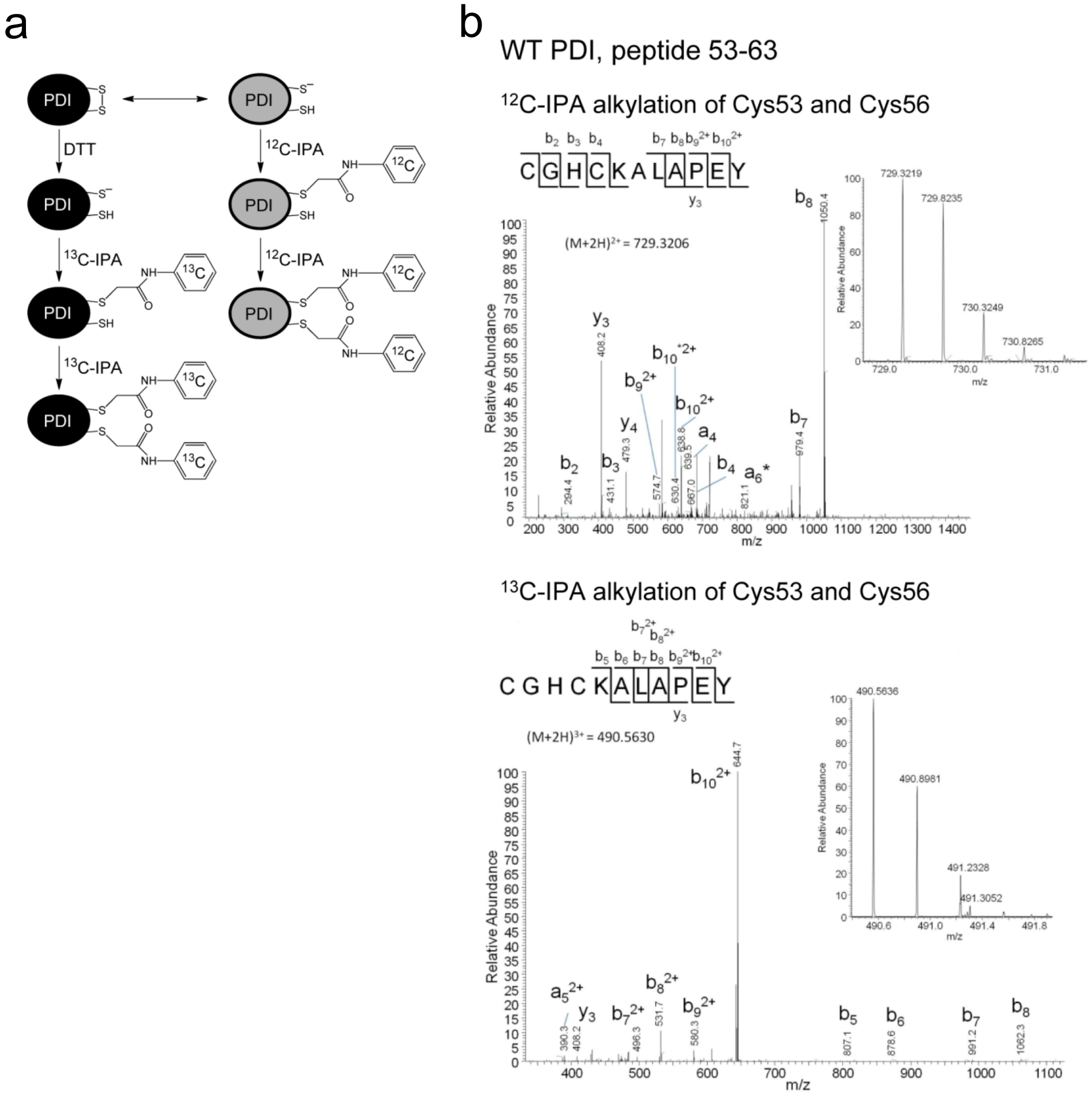

Supplementary Figure 9. Differential cysteine alkylation and mass spectrometry of PDI. (a) Reduced disulfide bond cysteines in PDI were alkylated with ^{12}C-IPA and the oxidized cysteine thiols with ^{13}C-IPA following reduction with DTT. One or both of the reduced disulfide bond cysteines can be labeled with either alkylator. The ratio of ^{12}C-IPA to ^{13}C-IPA alkylation represents the proportion of the disulfide bonds in the population that are in the reduced state. (b) Tandem mass spectrum of the WT PDI CGHCKALAPEY peptide containing the Cys53 and Cys56 catalytic dithiol/disulfide. The top panel is an example of the ^{12}C-IPA-alkylated peptide and the bottom panel the ^{13}C-IPA-alkylated peptide. Both Cys53 and Cys56 are alkylated in these examples. The accurate mass spectrum of the peptide is shown in the inset (observed [M+2H]2+ = 729.3219 m/z and expected [M+2H]2+ = 729.3207 m/z; observed [M+2H]3+ = 490.5636 m/z and expected [M+2H]2+ = 490.5630 m/z).

PDI	Active-site disulfide	$E^{o'}$, mV
No Addition	a (Cys53-Cys56)	-206 ± 37
	a' (Cys397-Cys400)	-206 ± 36
+ bepristat 1a	a (Cys53-Cys56)	-209 ± 60
	a' (Cys397-Cys400)	-210 ± 72
+ bepristat 2b	a (Cys53-Cys56)	-201 ± 48
	a' (Cys397-Cys400)	-200 ± 58

SupplementaryTable 1. Redox potential of PDI in the presence of bepristats. The calculated equilibrium constants based on results shown in **Fig. 5** were used to determine the standard redox potentials from equation 2 in Methods.

Supplementary Methods

P-Selectin expression

P-selectin expression, a marker of platelet activation, was monitored by flow cytometry. Washed human platelets (2.5 x 10^8 platelets / mL), prepared as described above, were incubated with 45 μM of bepristat 1b, 45 μM of bepristat 2a, or vehicle control. After exposure to 2 μM of SFLLRN, 5 μL of PE-conjugated CD62P (BD Biosciences) was added to 25 μL of the platelet sample. Fluorescence and forward scatter measurements were performed using a Gallios Flow Cytometer (Beckman Coulter). The data was analyzed using Kaluza software and Graphpad Prism 5.0.
